# Covariability of western tropical Pacific-North Pacific atmospheric circulation during summer

**DOI:** 10.1038/srep16980

**Published:** 2015-11-23

**Authors:** Kyung-Sook Yun, Sang-Wook Yeh, Kyung-Ja Ha

**Affiliations:** 1 Research Center for Climate Sciences, Pusan National University, Busan, South Korea; 2 Division of Earth Environmental System, College of Natural Science, Pusan National University, Busan, South Korea; 3 Department of Marine Sciences and Convergent Technology, Hanyang University, ERICA, South Korea

## Abstract

North Pacific subtropical high (NPSH) is permanent high-pressure system over the Northern Pacific Ocean and it extends to the western North Pacific during the boreal summer (June-July-August), which is so called the western North Pacific subtropical high (WNPSH). Here, we examine the covariability of the NPSH-WNPSH during summer using both observation and Coupled Model Intercomparison Project phase 5 (CMIP5) model data. The statistical analyses indicate that the NPSH-WNPSH covariability shows significant decadal variability in the observations, in addition, the in-phase relationship of NPSH-WNPSH is enhanced after the mid-to-late 1990s. A dipole-like sea surface temperature (SST) pattern, i.e., a warming in the western Pacific and a cooling in the eastern Pacific, is dominant after the mid-to-late 1990s, which acts to enhance the covariability of NPSH-WNPSH by modulating the atmospheric teleconnections. However, the covariability of NPSH-WNPSH in the future climate is not much influenced by the anthropogenic forcing but it is largely characterized by the natural decadal-to-interdecadal variability, implying that the enhancement of NPSH-WNPSH covariability after the mid-to-late 1990s could be considered as part of decadal-to-interdecadal variability.

North Pacific subtropical high (NPSH), which is a fundamental element of atmospheric circulation over the Northern Pacific Ocean, extends to the western North Pacific during boreal summer^1^ (Fig. 1A, and see Supplementary Information). The westward extension of NPSH is referred as to the western North Pacific subtropical high (WNPSH). The origin and influence on weather and climate variability of these subtropical highs display differences and similarities^2^,^3^,^4^,^5^,^6^,^7^. Whereas the formation of the NPSH can be explained by local topography, monsoonal heating, local atmosphere-ocean interaction, and near-surface thermal contrast between the land and ocean^8^,^9^,^10^, the WNPSH is caused mainly by positive thermodynamic feedback between the ocean and atmosphere in Indian Ocean and western Pacific (IO-WP) warm pool^11^,^12^. The WNPSH links the extratropical summer monsoon system over East Asia to tropical sea surface temperature (SST) and El Niño-Southern Oscillation (ENSO) forcing^12–14^, and hence acts as a regulator of weather and climate variability over East Asia^2^,^15^,^16^. On the other hand, the variability of NPSH modulates the weather and climate over the northeastern Pacific and North America^6^,^7^. Despite these differences, however, both the NPSH and WNPSH play key roles in the transport of momentum and energy between the tropics and extratropics, respectively.

Significant changes in the relationship between tropical SSTs and subtropical highs over the North Pacific have recently been detected^17^. Concurrently, the Coupled Model Intercomparison Project (CMIP) phase 3 model simulations suggest an intensification of the NPSH as a result of the enhanced thermal land-sea contrast in the future climate with an increase of greenhouse gas^18^. Furthermore, while these subtropical highs are correlated with each other during the last three decades (*r* ∼ 0.50 during 1979–2010, where *r* is a correlation coefficient), the historical relationship between NPSH and WNPSH is insignificant (*r* ∼ 0.1 during 1920–1978) (Supplementary Fig. S1 and Fig. 2B), implying the multi-decadal change in the interannual covariability between NPSH and WNPSH. These results raise important questions as to whether the characteristic relationship in the two subtropical highs has been altered and how such a relationship would change under global warming. Here we examine the characteristics of covariability between NPSH and WNPSH using the observations and the CMIP phase 5 (CMIP5)^19^ model data.

## Results

### Recent enhancement of covariability in two subtropical high-pressures

**Figure 1:**
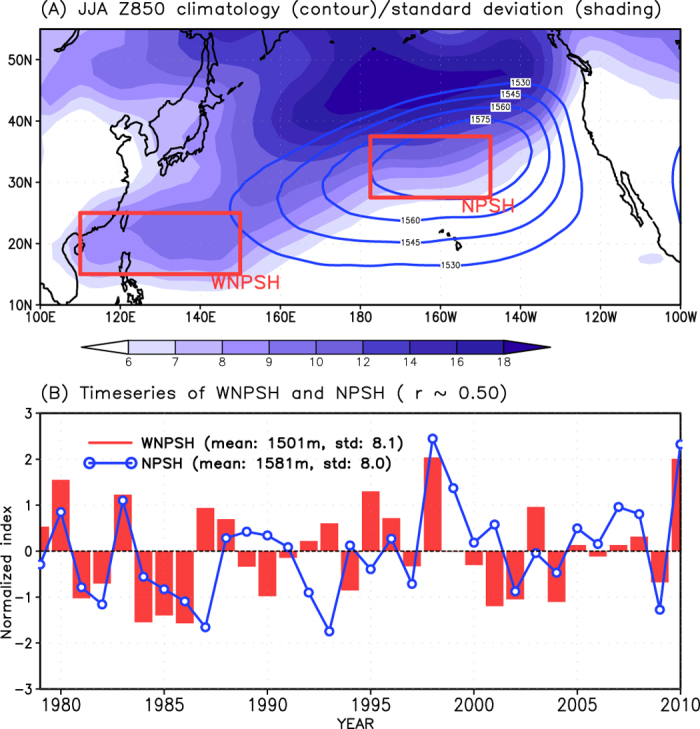
Summertime NPSH and WNPSH. (**A**) Climatology (contour) and interannual standard deviation (shading) of June-July-August (JJA) geopotential height at 850 hPa (Z850) obtained from from the National Centers for Environmental Prediction–Department of Energy (NCEP2) Reanalysis during 1979–2010. Red boxes represent the domains in western North Pacific subtropical high (WNPSH) and North Pacific subtropical high (NPSH). (**B**) Interannual variability of summertime NPSH and WNPSH. The map in this figure was drawn using GrADS.

**Figure 2:**
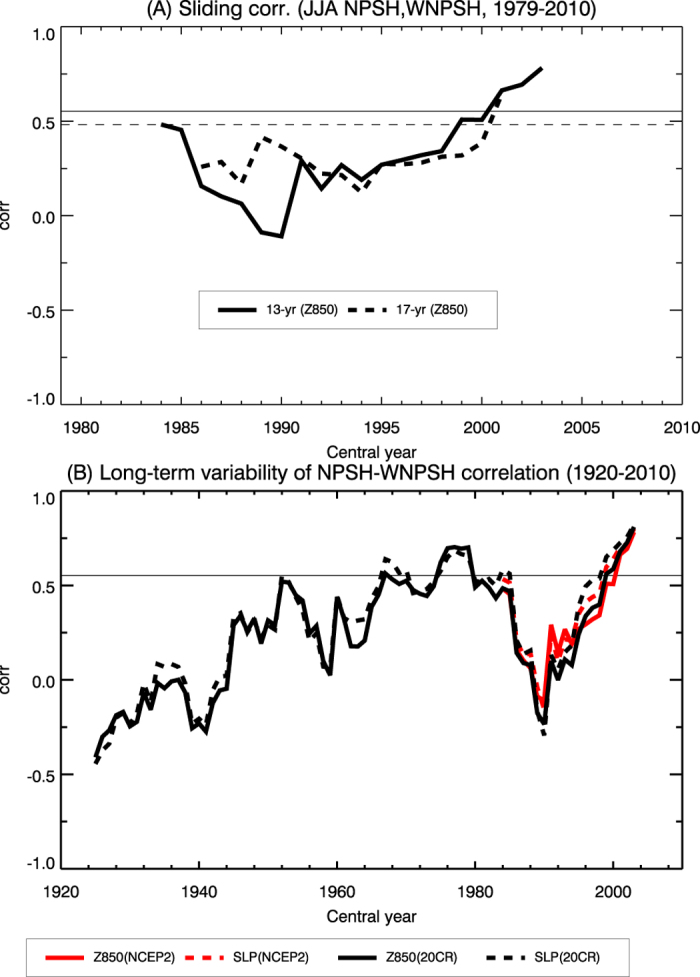
Enhanced covariability between NPSH and WNPSH. (**A**) Sliding correlation coefficient between the JJA WNPSH and NPSH during 1979–2010 with 13-yr (solid line) and 17-yr (dashed line) windows. (**B**) Long-term variability of the NPSH-WNPSH correlation coefficient with 13-yr window during 1920–2010. The horizontal solid (dashed) line indicates the correlation coefficient significant at the 95% confidence level with 13-yr (17-yr) window. The subtropical high indices are calculated using Z850 and sea level pressure (SLP) from twentieth Century Reanalysis version 2 (20CR) during 1920–2010 and NCEP2 during 1979–2010.

We first show the change in the relationship between NPSH and WNPSH using the seasonal (June-July-August; JJA) mean 850 hPa geopotential height (Z850) anomaly for the period 1979–2010. The sliding correlation coefficients between the intensities of the WNPSH and NPSH reveal a gradual increase with decadal fluctuation (Fig. 2A). Further analysis using Z850 and sea level pressure (SLP) anomalies for 1920–2010 also confirms that the covariability of the two subtropical high-pressures has been strengthened along with significant decadal variabilities (Fig. 2B). It should be noted that there exist no statistically significant increasing trends of NPSH and WNPSH in the observations (see Fig. 1B and Supplementary Fig. S1).

**Figure 3:**
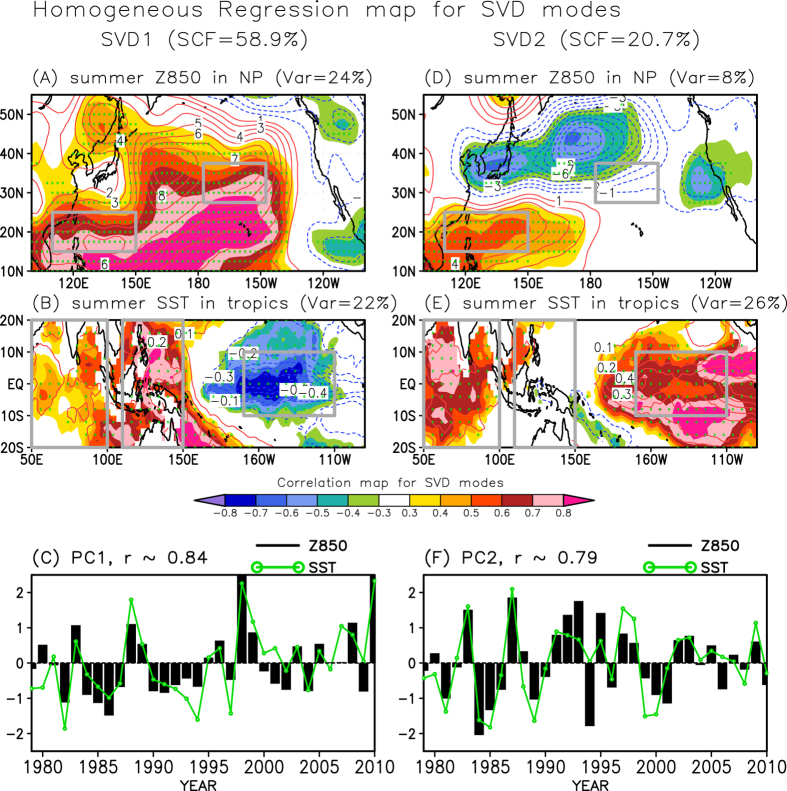
Relationship between tropical SST and North Pacific subtropical high. (**A**, **B**) Homogeneous regression map (red and blue contours) against the first singular value decomposition (SVD1) mode between (**A**) the summer North Pacific Z850 from NCEP2 and (**B**) the summer tropical sea surface temperature (SST) from the Met Office Hadley Centre Sea Ice and Sea Surface Temperature (HadISST) for the period 1979–2010. (**D**, **E**) Same as (**A**, **B**), but for the SVD2 mode. Shading indicates the homogeneous correlation map for each SVD mode and the green dots denote the regressed values significant at the 95% confidence level. SCF is the squared covariance fraction explained by the SVD mode and Var is the variance fraction explained by the SVD Z850 and SST pattern. The domains interested in this study are denoted by gray boxes. (**C**,**F**) The associated (**C**) first and (**F**) second principal component (PC) time series for 1979–2010. The map in this figure was drawn using GrADS.

Figure 2 indicates that there exists the interdecadal change in the covariability of WNPSH-NPSH on interannual timescales. Our hypothesis is that this interdecadal change in NPSH-WNPSH covariability could be related to variability of tropical SSTs covering from IO-WP to eastern Pacific (EP). Previous studies have suggested that the tropical SST forcing plays an important role in modulating both the WNPSH and NPSH variability^3^,^11^,^17^. To examine the statistical connection between tropical SSTs and NPSH-WNPSH covariability, we perform the singular value decomposition (SVD) analysis of the JJA mean SST and the Z850 for the period 1979–2010. The first two SVD modes (i.e., SVD1 and SVD2) explain approximately 80% of the total squared covariance between SST and Z850 (Fig. 3). The first two principal component (PC) time series of SST correlate strongly with those of Z850 (*r* ∼ 0.84 for SVD1 and *r* ∼ 0.79 for SVD2), verifying a critical role of the tropical SST variability on the NPSH-WNPSH covariability. Furthermore, the SST variability due to the SVD1 and SVD2 explains substantial variance of 58% (42%) for NPSH (WNPSH).

The spatial structure of the SVD1 and its PC time series indicate that an in-phase relationship of the NPSH and WNPSH is associated with a dipole-like SST pattern, i.e., a warming (cooling) in the IO-WP and a cooling (warming) in the EP (Fig. 3A,B; Supplementary Figs S2A and S2B). Note that the SVD1 result excluding the effect of IO SST reveals an identical in-phase relationship between NPSH and WNPSH (see Supplementary Fig. S3), showing that the impact of IO SST on the in-phase covariability of subtropical high is less significant than that of the WP and EP SSTs. The positive dipole SST pattern between WP warming and EP cooling is more prominent after the mid-to-late 1990s (Fig. 3C), which might be related to the increased covariability between NPSH and WNPSH.

On the other hand, the spatial structure of the SVD2 reflects an out-of-phase height variance over North Pacific, which is associated with the triple-like structure in the tropical basins (Fig. 3D,E; Supplementary Figs S2C and S2D). The triple-like pattern of IO warming, WP cooling, and EP warming is related to a meridional shrinking of NPSH and strengthening of WNPSH. The difference in intensity between WNPSH and NPSH is significantly correlated with the SVD2 time series of SST and Z850 (*r* ∼ 0.54 in SST and *r* ∼ 0.74 in Z850), which indicates that the SVD2 represents an out-of-phase in the two subtropical high-pressures. Recent studies have effectively described how the IO warming and WP cooling generate the meridional dipole height pattern over western North Pacific through positive atmosphere-ocean feedback processes^3^,^11^. This implies that the SVD2 mode of variability is mainly associated with an intrinsic air-sea coupled system (see Supplementary Information). In particular, the variability of SVD2 PC time series has been reduced after the mid-to-late 1990s (Fig. 3F), which also provides a favorable condition for strengthening of the covariability of NPSH-WNPSH.

**Figure 4:**
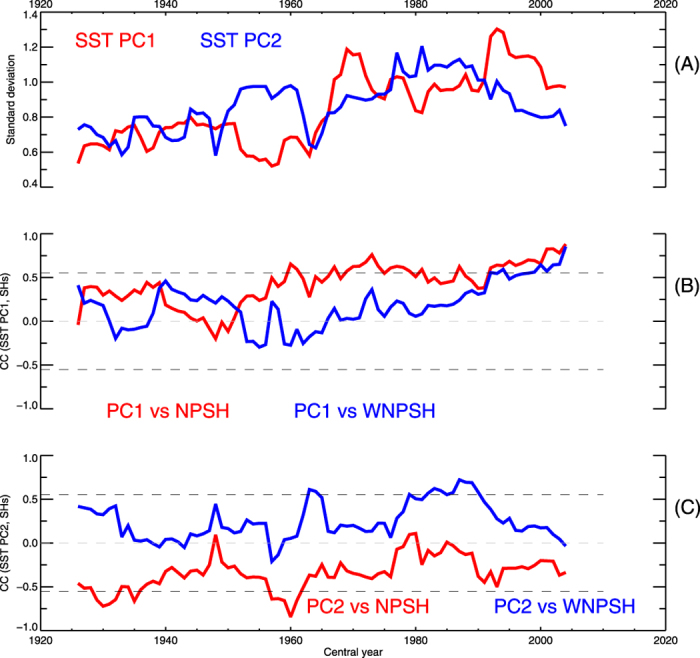
Change in the relationship between tropical SSTs and subtropical highs. (**A**) 13-yr window sliding standard deviation of projected SST time series onto the SST pattern of SVD1 and SVD2 during 1920–2010. (**B**) 13-yr window sliding correlation coefficient between the projected SST time series of SVD1 and JJA subtropical highs. (**C**) Same as (**B**), but for the projected SST time series of SVD2. The horizontal black dashed line indicates the value significant at the 95% confidence level. The high indexes are calculated using Z850 from 20CR during 1920–2010.

How a dipole-like SST pattern in the tropical basins, i.e., a warming (cooling) in the WP and a cooling (warming) in the EP, acts to enhance the covariability of NPSH-WNPSH after the mid-to-late 1990s? To examine this statistically, we display the 13-yr sliding standard deviation of the SST time series projected onto SST pattern of SVD1 and SVD2 during 1920–2010, respectively (Fig. 4A). While the variability of a dipole-like SST pattern (i.e., SVD1) shows a considerable enhancement, that of tri-polar SST pattern (i.e., SVD2) is reduced after the mid-to-late 1990s. Overall, the NPSH is strongly correlated with the WNPSH under the condition that the variability of SVD1 is larger than that of SVD2 (see Figs 2B and 4A). Furthermore, the dipole-like SST pattern of SVD1 is significantly connected with the both NPSH and WNPSH after the mid-to-late 1990s (Fig. 4B). The triple SST pattern of SVD2 tends to be related to the opposite sign between NPSH and WNPSH (Fig. 4C). Note that the interdecadal change of the NPSH-WNPSH covariability arises from the change in relative dominance between SST patterns of SVD1 and SVD2, which is associated with the changes in relationship of each basin of tropical SSTs (Supplementary Fig. S4). The connection of subtropical highs with SST pattern of SVD2 seems to be weakened after the mid-to-late 1990s, which is significantly attributable to the variability of IO SST (see Supplementary Fig. S4D). In contrast, the connection of subtropical highs with SST pattern of SVD1 is contributed by both WP and EP SST variability (Supplementary Figs S4B and S4C). This result statistically provides the recent strengthening of dipole-like SST with the covariability of NPSH-WNPSH.

### Covariability under future global warming

**Figure 5:**
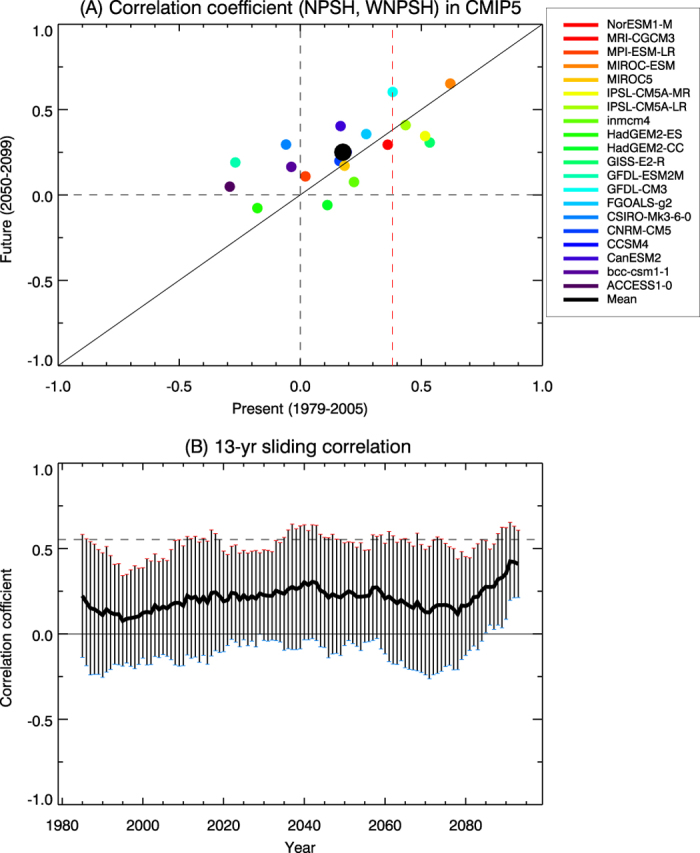
Covariability change in the CMIP5 future climate simulation. (**A**) Scatter plot of correlation coefficients between NPSH and WNPSH of Z850 simulated by 20 Coupled Model Intercomparison Project Phase 5 (CMIP5) individual models: (x-axis) the present climate of 1979–2005 in historical run; (y-axis) the future climate of 2050–2099 in RCP 4.5 run. The black filled circle denotes the averaged correlation coefficient for all CMIP5 models and the vertical red dashed line indicates the value significant at the 95% confidence level. (**B**) Multi-model average of 13-yr window sliding correlation coefficient between NPSH and WNPSH for all CMIP5 models during 1979–2099. The vertical error bar indicates one standard deviation of inter-model variability.

The IO-WP SST (20°S-20°N, 50°E-160°E) has warmed by 0.39 °C during the last 32 years (Supplementary Fig. S5). Such rapid warming may be associated with radiative forcing due to an increase in greenhouse gases concentration^20^,^21^, which raises an important question as to how the covariability of NPSH-WNPSH is changed under future global warming. To examine this issue, we use CMIP5 model data in the historical run for the period of 1979–2005 and the Representative Concentration Pathway (RCP) 4.5 run for the period of 2006–2099. Figure 5A shows the correlation coefficient between NPSH and WNPSH from the present climate (1979–2005) to the future climate (2050–2099) using all CMIP5 climate models. It is evident that the covariability of WNPSH-NPSH is quite diverse and there exist no significant changes of NPSH-WNPSH covariability from the present climate to the future climate: increase in 13 models and decrease in 7 models. The CMIP5 models represent a considerable range of NPSH-WNPSH covariability on interdecadal timescales for 1979–2099 with its inter-model spread (Fig. 5B). Supplementary Figure S6 also reveals that the covariability of NPSH-WNPSH is characterized by decadal fluctuations from the present to the future climate in the CMIP5 climate models, which is consistent with the result using SLP (Supplementary Fig. S7). This indicates that the covariability of WNPSH-NPSH in the future climate is not much influenced by the anthropogenic forcing but it might be largely associated with the natural variability.

## Discussion

Coherent statistical evidences suggest the recent intensification of the covariability between NPSH and WNPSH along with considerable decadal variabilities. The strengthening of covariability in the two subtropical highs after the mid-to-late 1990s is associated with the dipole-like SST pattern in the tropical basins, i.e., a warming (cooling) in the WP and a cooling (warming) in the EP. The CMIP5 future climate simulation shows no significant change in the relationship of NPSH-WNPSH covariability. These reflect that a considerable portion in strengthening of covariability in the two subtropical highs in the observations is derived from the natural variability on multi-decadal timescales.

We speculate that the interdecadal change in covariability of WNPSH-NPSH is significantly associated with the change in the air-sea interactions in the WP such as the SST-convective forcing. For example, the convective response to the anomalous SST forcing in the WP is quite different before and after the mid-to-late 1990s (see Supplementary Fig. S8). After the mid-to-late 1990s, the extensive convective forcing over the WP region would induce northeasterly flow and divergence over the WNP via a baroclinic Kelvin wave into Pacific^22^, resulting in the suppressed convection and anomalous anticyclone over the WNP. Meanwhile, the cooling SST in EP also contributes to the suppressed convection via modulation of the east-west Walker circulation^3^. Therefore, the coupled feature of the warming (cooling) in WP and the cooling (warming) in EP can serve to strengthen the in-phase covariability of the NPSH-WNPSH by modulating the atmospheric teleconnections^17^,^23^,^24^. Note that the convective response on the SST anomalies in WP is restricted to the WP region and it generates enhanced convection over the WNP before the mid-to-late 1990s. The detailed dynamics are not firmly demonstrated yet. It is necessary to conduct the numerical experiments using the climate models to isolate the role of WP warming on the covariability of NPSH-WNPSH.

## Methods

### Observation datasets

The present study used monthly mean SST from the Met Office Hadley Centre Sea Ice and Sea Surface Temperature version 1.1 (HadISST1.1)^25^ for the period 1920–2010 and atmospheric circulation variables from the National Centers for Environmental Prediction–Department of Energy (NCEP–DOE; NCEP2)^26^ for the period 1979–2010. To support the present conclusion with a longer-period, we also used the atmospheric data obtained from the twentieth Century Reanalysis version 2 (20CR)^27^ for the period 1920–2010.

The Z850 and SLP were used to identify the NPSH and WNPSH. The NPSH and WNPSH indices were calculated as the area-averaged anomalies of Z850 and SLP over [27.5°N−37.5°N, 177.5°W−147.5°W] and [15°N−25°N, 110°E−150°E]. To investigate the major coupled patterns between tropical SSTs and mid-latitude North Pacific, we applied a SVD analysis to the JJA seasonal mean tropical SST and extratropical North Pacific atmospheric system, i.e., IO-WP-EP SST anomaly over [20°S–20°N, 50°E–90°W] and North Pacific geopotential height anomaly at 850 hPa over [10°N–55°N, 100°E–100°W]. The statistical significance for the composite and regression analysis was estimated with a two-tailed Student’s t test with the degree of freedom of n−2.

### CMIP5 model data

To investigate future changes of tropics-midlatitudes atmospheric covariability under anthropogenic global warming, we used the 20 coupled general circulation models (CGCMs) that participated in CMIP5. The used model, institution and horizontal resolution of the atmospheric components are represented in Supplementary Table S1. To focus on the interannual covariability, we use only one ensemble member for the historical and RCP4.5 runs in individual models. Two experiments were adopted in this study including the historical run from 1850 to 2005 and the RCP 4.5 run from 2006 to 2100. For present climate projection, changing conditions consistent with observations, such as CO_2_ due to both anthropogenic and natural forcing, were imposed on the historical run. The RCP4.5 run, in which radiative forcing stabilized at ~4.5 Wm^−2^ after 2100, was chosen for future climate projection^8^. We analyzed the present climate for the period of 1979 to 2005 and the future climate for the 21^st^ century (i.e., 2006–2099).

## Additional Information

**How to cite this article**: Yun, K.-S. *et al.* Covariability of western tropical Pacific-North Pacific atmospheric circulation during summer. *Sci. Rep.* **5**, 16980; doi: 10.1038/srep16980 (2015).

## Supplementary Material

Supplementary Information
