# Supplementary material for: Covariability of western tropical Pacific-North Pacific atmospheric circulation during summer
Source: Sci Rep. 2015 Nov 23;5:16980. doi: 10.1038/srep16980 (PMC4655338; doi:10.1038/srep16980)
Supplement: Supplementary Information [file srep16980-s1.doc]

**Supplementary Information**

**Covariability of western tropical Pacific-North Pacific atmospheric circulation during summer**

Kyung-Sook Yun^1^, Sang-Wook Yeh^3^, and Kyung-Ja Ha^1, 2*^

^1^Research Center for Climate Sciences, Pusan National University, Busan, South Korea

^2^Division of Earth Environmental System, **College of Natural Science,**

Pusan National University, Busan, South Korea

^3^Department of Marine Sciences and Convergent Technology, Hanyang University, ERICA, South Korea

**NPSH and WNPSH**

The North Pacific subtropical high (NPSH) is permanent subtropical anticyclone occurring over nearly entire North Pacific Ocean. The western North Pacific subtropical high (WNPSH) is anomalously generated by the southwestward extension of the summertime NPSH. We identified the WNPSH and NPSH by using the summertime (June-July-August) climatology and interannual variability of 850 hPa geopotential height (hereafter, Z850; unit is m). As shown in Fig. 1A, the NPSH is prominently displayed in the summertime 850 hPa height climatology, while the WNPSH is obvious only in the interannual variability, indicated by year to year standard deviation. The NPSH and WNPSH indices were calculated as the area-averaged anomalies of Z850 over [27.5°N−37.5°N, 177.5°W−147.5°W] and [15°N−25°N, 110°E−150°E], represented in Fig. 1A by red boxes. The domains of NPSH and WNPSH were roughly consistent with those in the previous studies (e.g., Li et al., 2012; Wang et al., 2013; Yun et al., 2013). In spite of using the NPSH and WPNSH indices defined in different regions, we obtain similar results as in Fig. 2A (Fig. S9).

In addition to the Z850, we define the WNPSH and NPSH indices using the surface level pressure (hereafter, SLP; unit is hPa). The interannual variability of NPSH is significantly connected with that of the WNPSH. The correlation coefficient between two highs during 1979-2010 was 0.50 in Z850 and 0.57 in SLP.

As shown in Fig. S10, most of the Coupled Model Intercomparison Project phase 5 (CMIP5) models reasonably simulate the mean structure of the NPSH, whereas several models display relatively weak WNPSH variability. For example, WNPSH variability in the INM-CM4, IPSL-CM5A-LR, and IPSL-CM5A-MR models is weaker than those in other models and observations.

**The coupled mode between tropical SST and subtropical high**

To identify the coupled mode associated with decadal climate variation, we performed SVD analysis of the three-year running mean SST and Z850 (Fig. S11). The SVD1 mode shows a similar dipole SST pattern (WP SST warming and EP cooling) and in-phase relationship of NPSH-WNPSH with those shown in Fig. 3. However, the SVD2 mode does not exhibit the WNPSH variability and presents a midlatitude disturbance-like wave structure, which is different from that in Fig. 3. The result suggests that the SVD2 mode in Fig. 3 is rooted in internal dynamics associated with interannual variability of WNPSH.

**Validation of observation datasets**

Reconstruction of SVD PC timeseries was conducted to test the observed initial SVD1 timeseries by using additional observation datasets, which includes NCEP (Kalnay et al., 1996) and European Centre for Medium-Range Weather Forecasts Reanalysis (ERA) data (Dee et al., 2011). Here, ERA (i.e., ERA40-interim) is composed of ERA40 (Uppala et al., 2005) from 1958 to 1978 and ERA-INTERIM from 1979 to 2010. All timeseries show significant increasing trends (Fig. S12).

According to previous studies (Hines et al., 2000; Trenberth et al., 2001; Wu and Xie 2003; Bromwich and Fogt, 2004; Wu et al., 2005), there exist serious problems before 1979 among reanalysis datasets, causing the misinterpretation on the changes in the atmospheric circulations. This is the reason why the periods of observations data presented in this paper are limited after 1979. To support the present conclusion with a longer-period, we have performed the same analyses using the atmospheric data obtained from the twentieth Century Reanalysis version 2 (20CR, Compo et al., 2011) during 1920-2010. We obtain similar results comparable to those in the NCEP2 observation during 1979-2010 (i.e., Figs. S1 and 2B).

**Reference**

Bell, G. D., & Janowiak, J. E. Atmospheric circulation associated with the Midwest floods of 1993. *Bull. Amer. Meteor. Soc.* **76**, 681-695 (1995).

Bromwich, D. H., & Fogt, R. L. Strong Trends in the Skill of the ERA-40 and NCEP–NCAR Reanalyses in the High and Midlatitudes of the Southern Hemisphere, 1958–2001. *J. Clim.* **17**, 4603-3619 (2004).

Dee, D. P., *et al.* The ERA-Interim reanalysis: configuration and performance of the data assimilation system. *Q. J. R. Meteorol. Soc.* **137**, 553–597 (2011).

Hines, K. M., Bromwich, D. H., & Marshall, G. J. Artificial surface pressure trends in the NCEP-NCAR reanalysis over the southern ocean and Antarctica. *J. Clim.* **13**, 3940-3952(2000).

Kalnay. E., *et al.* The NCEP/NCAR 40-year reanalysis project. *Bull. Am. Meteorol. Soc.* **77**, 437–71 (1996).

Lau, K.-M., Lee, J.-Y., Kim, K.-M., & Kang, I.-S. The north Pacific as a regulator of summertime climate over Eurasia and North America. *J. Clim.* **17**, 819-833 (2004).

Li, W., Li, L., Ting, M., & Liu, Y. Intensification of northern Hemisphere subtropical highs in a warming climate. *Nature Geosci.* **5**, 830-834, doi:10.1038/nego1590 (2012).

Trenberth, K. E., Stepaniak, D. P., & Hurrell, J. W. Quality of Reanalyses in the Tropics. *J. Clim.* **14**, 1499-1510 (2001).

Uppala, S. M., *et al.* The ERA-40 re-analysis. *Q. J. R. Meteorol. Soc.* **131**, 2961–3012 (2005).

Wang, B., Xiang, B., & Lee, J.-Y. Subtropical high predictability establishes a promising way for monsoon and tropical storm predictions. *Proc. Natl. Acad. Sci. U. S. A.* **110**(8), 2718-2722 (2013).

Wu, R., & Xie, S.-P. On equatorial Pacific surface wind changes around 1977: NCEA-NCAR reanalysis versus COADS observations. *J. Clim.* **16,** 167-173 (2003).

Wu, R., Kinter, J. L., & Kirtman, B. P. Discrepancy of interdecadal changes in the Asian region among the NCEP-NCAR reanalysis objective analyses and observations. *J. Clim.* **18**, 3048-3067 (2005).

**Table S1.** Description of CMIP5 models used in the study.

| **Coupled Model** | **Institution** | **AGCM**  **Resolution** |
| --- | --- | --- |
| **ACCESS1-0** | Commonwealth Scientific and Industrial Research Organisation and Bureau of Meteorology, Australia (CSIRO-BOM) | 1.875^o^x1.25^o^ |
| **BCC-CSM1.1** | Beijing Climate Center, China Meteorological Administration (BCC) | 2.8125^o^x2.8125^o^ |
| **CanESM2** | Canadian Centre for Climate Modelling and Analysis (CCCma) | 2.8125^o^x2.8125^o^ |
| **CCSM4** | National Center for Atmospheric Research (NCAR) | 1.25^o^x0.9375^o^ |
| **CNRM-CM5** | Centre National de Recherches Meteorologiques / Centre Europeen de Recherche et Formation Avancees en Calcul Scientifique  (CNRM-CERFACS) | 1.40625^o^x  11.40625^o^ |
| **CSIRO-Mk3-6-0** | Commonwealth Scientific and Industrial Research Organisation and the Queensland Climate Change Centre of Excellence (CSIRO-QCCCE) | 1.875^o^x1.875^o^ |
| **FGOALS-g2** | LASG, Institute of Atmospheric Physics, Chinease Academy of Sciences; and CESS, Tsinghua University (LASG-CESS) | 2.8125^o^x2.8125^o^ |
| **GFDL-CM3** | Geophysical Fluid Dynamics Laboratory  (NOAA GFDL) | 2.5^o^x2^o^ |
| **GFDL-ESM2M** |  | 2.5^o^x2^o^ |
| **GISS-E2-R** | NASA Goddard Institute for Space Studies  (NASA GISS) | 2.5^o^x2^o^ |
| **HadGEM2-CC** | Met Office Hadley Centre (MOHC) | 1.875^o^x1.24^o^ |
| **HadGEM2-ES** |  | 1.875^o^x1.24^o^ |
| **INM-CM4** | Institute for Numerical Mathematics (INM) | 2^o^x1.5^o^ |
| **IPSL-CM5A-LR** | Institute Pierre-Simon Laplace (IPSL) | 3.75^o^x1.875^o^ |
| **IPSL-CM5A-MR** |  | 2.5^o^x1.258^o^ |
| **MIROC5** | Atmosphere and Ocean Research Institute (University of Tokyo), National Institute for Environmental Studies, and Japan Agency for Marine-Earth Science and Technology (MIROC) | 1.40625^o^x  1.40625^o^ |
| **MIROC-ESM** |  | 2.8125^o^x2.8125^o^ |
| **MPI-ESM-LR** | Max Planck Institute for Meteorology (MPI-M) | 1.875^o^x1.875^o^ |
| **MRI-CGCM3** | Meteorological Research Institute (MRI) | 1.125^o^x2.25^o^ |
| **NorESM1-M** | Norwegian Climate Centre (NCC) | 2.5^o^x1.875^o^ |


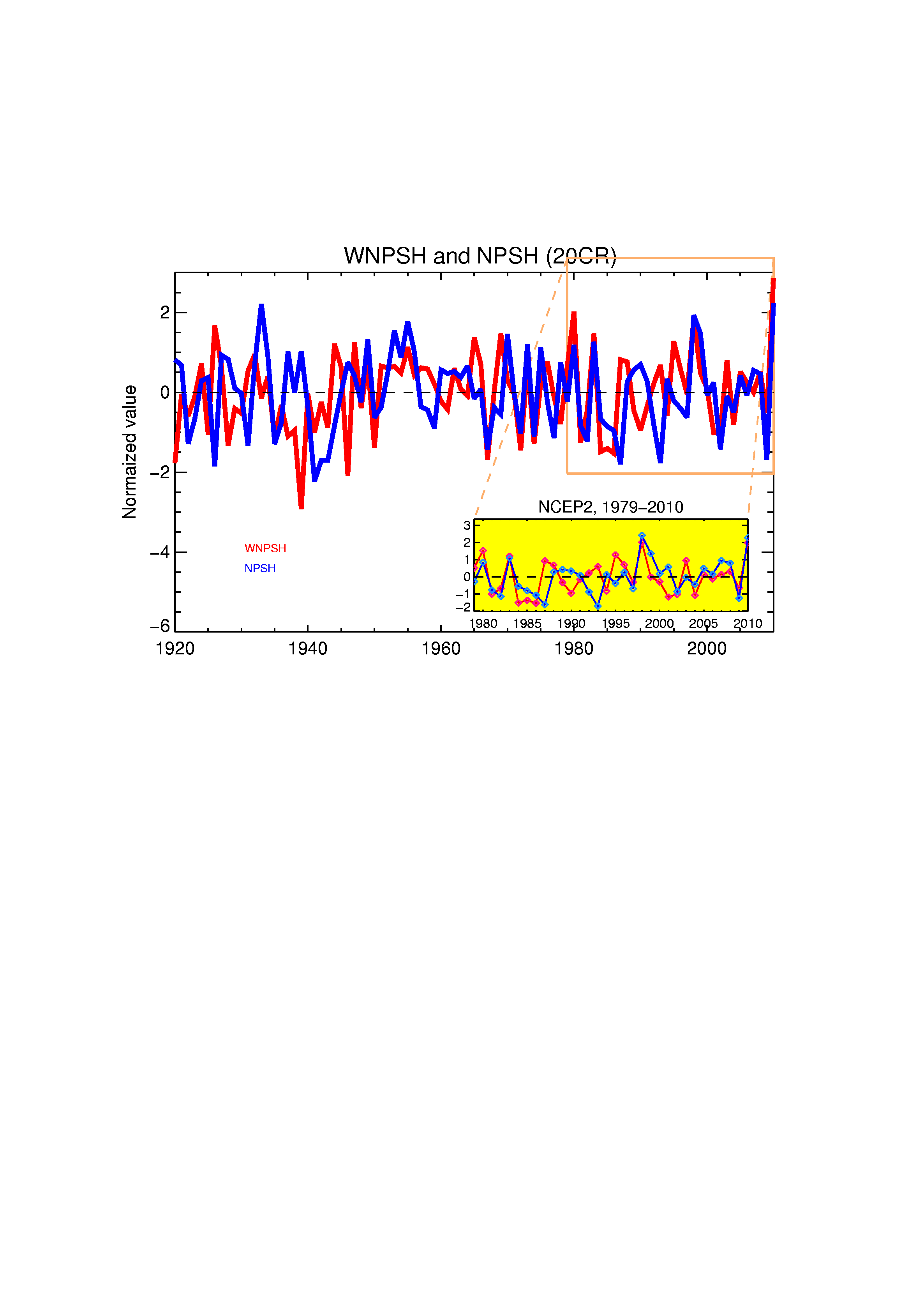


**Figure S1.** Normalized indices of WNPSH (red line) and NPSH (blue line) using the Z850 obtained from 20CR data during 1920-2010 and NCEP2 data during 1979-2010 (in box plot).


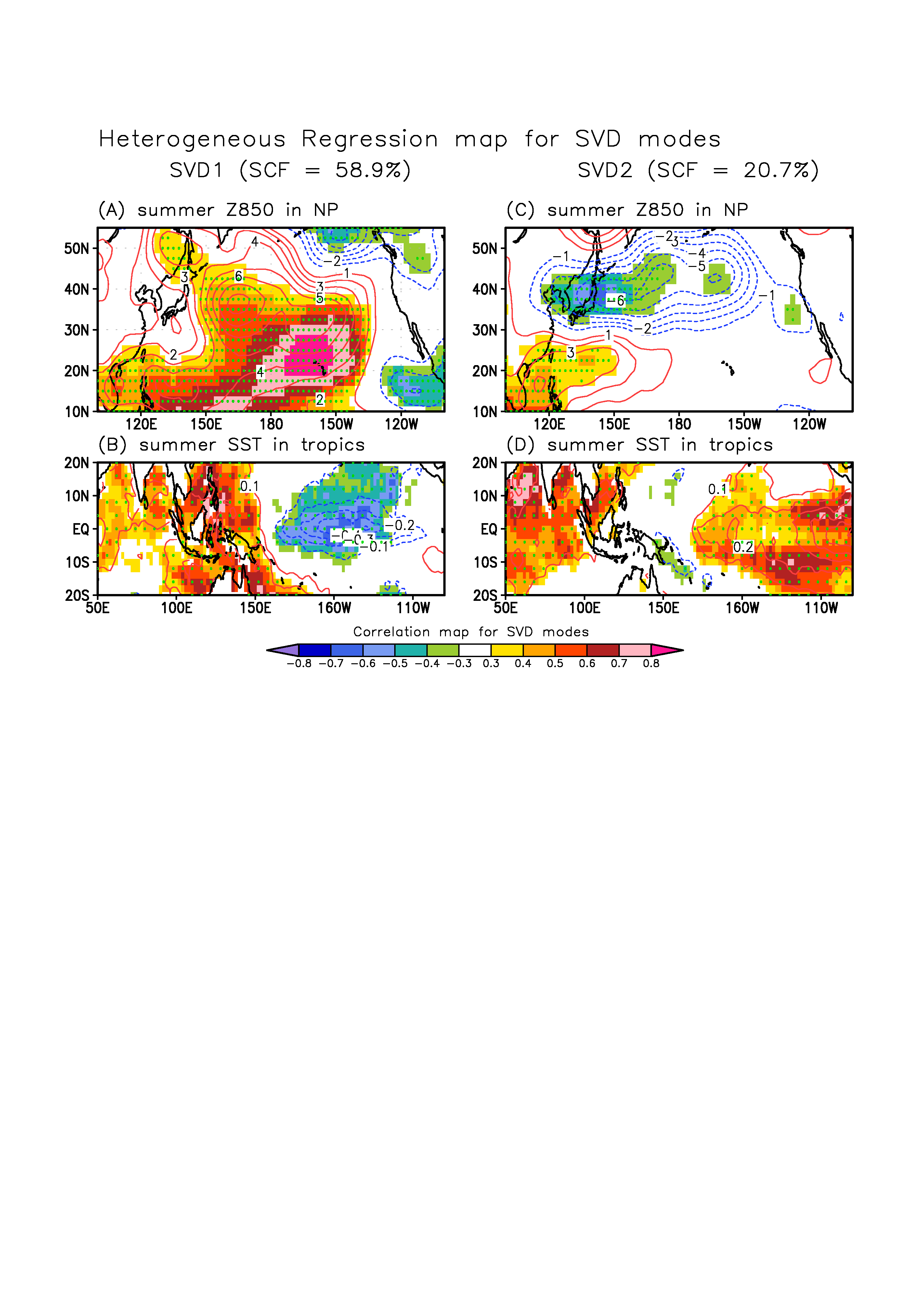


**Figure S2.** Same as Fig. 3, except for the heterogeneous regression. The map in this figure was drawn using GrADS.


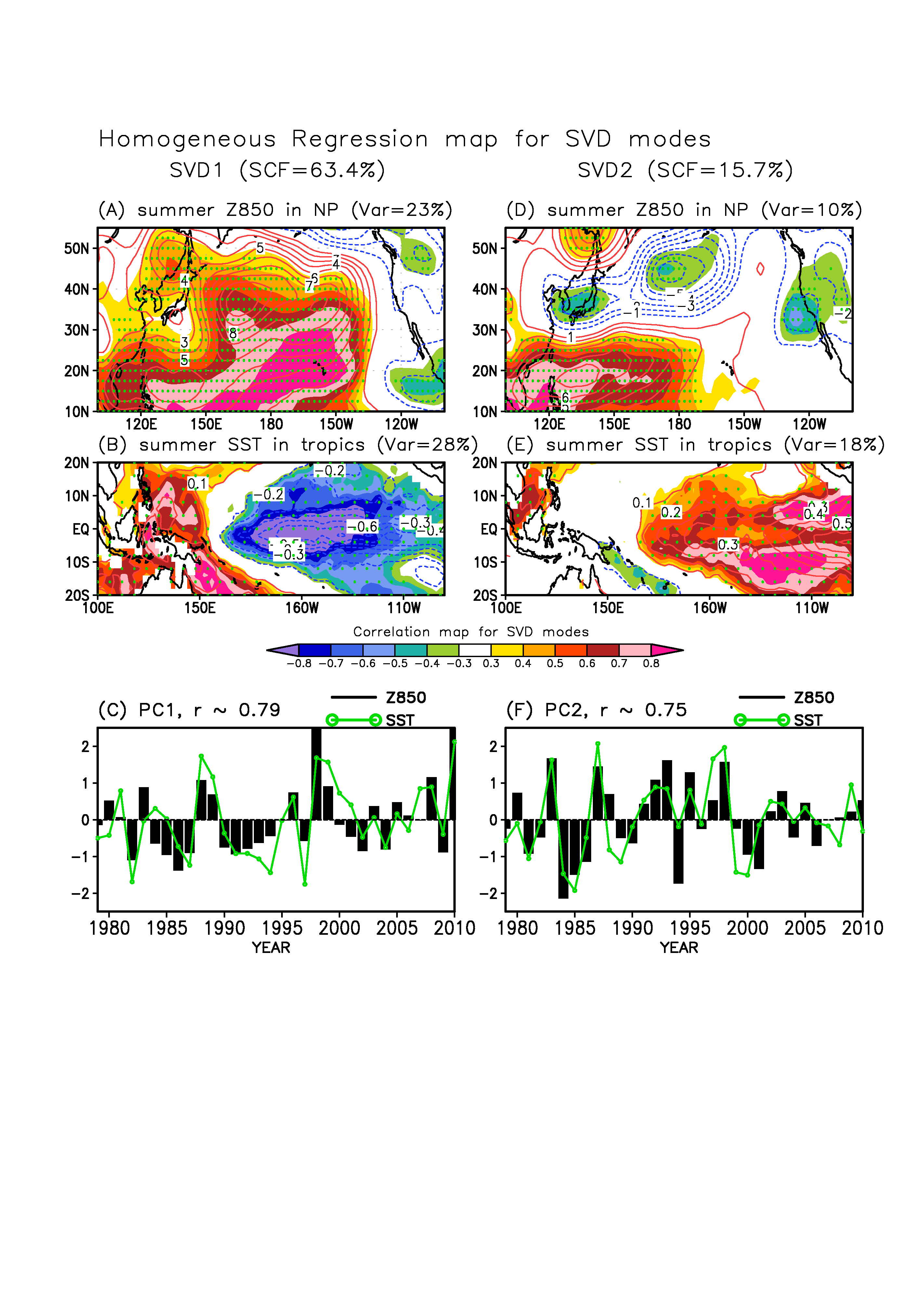


**Figure S3.** Same as Fig. 3, but for the SST domain over Pacific Ocean [20°S-20°N, 100°E-90°W]. The map in this figure was drawn using GrADS.


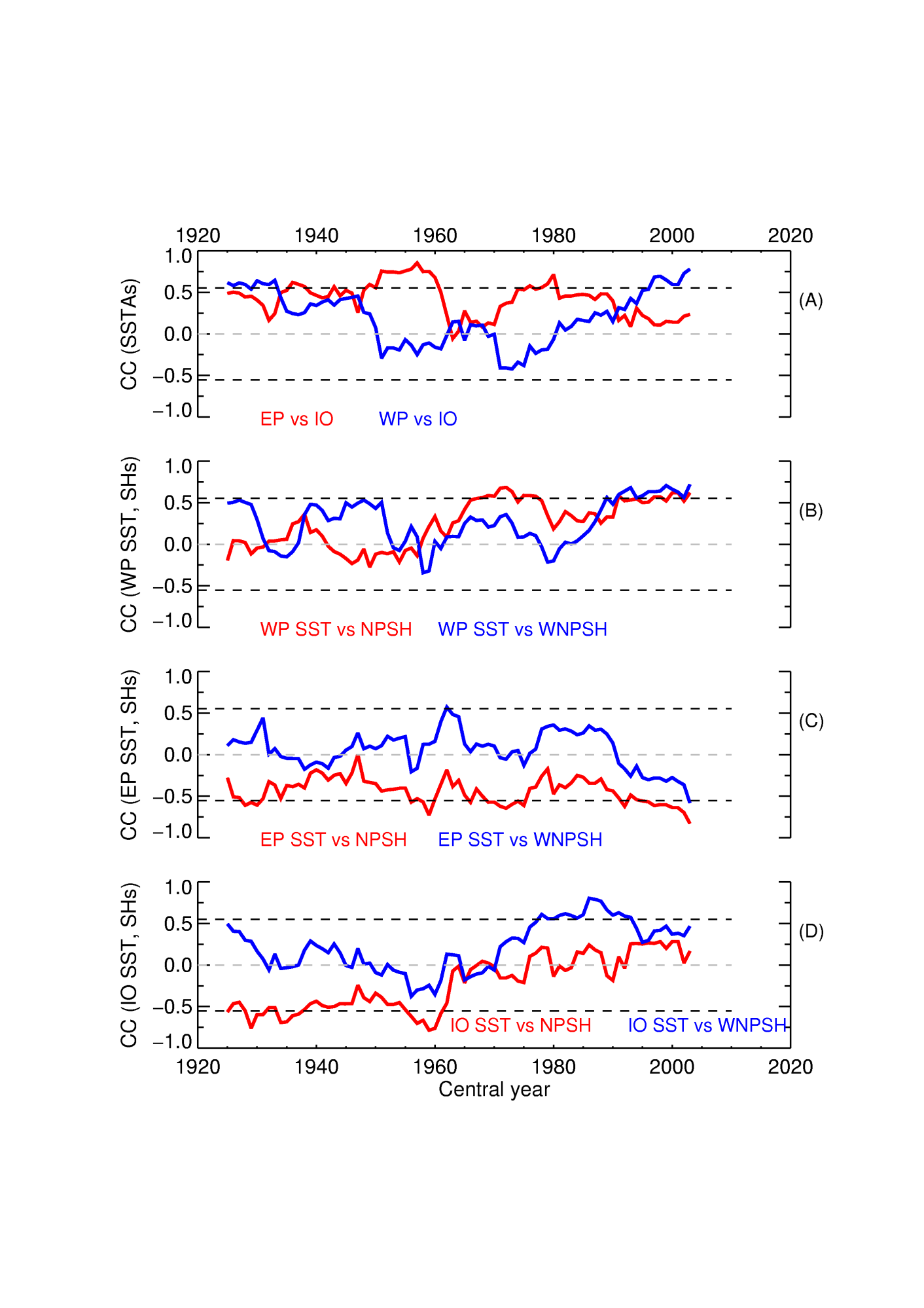


**Figure S4.** (A) 13-yr window sliding correlation coefficient between the JJA tropical SSTs from HadISST averaged over the domains: Indian Ocean [IO; 20°S-20°N, 50°E-100°E]; western Pacific [WP; 20°S-20°N, 110°E-150°E]; eastern Pacific [EP; 10°S-10°N, 170°W-110°W]. The defined domains are denoted by the gray boxes in Figs. 3B and 3E. The horizontal black dashed line indicates the value significant at the 95% confidence level. Same as (A), but for correlation coefficient between the subtropical highs and (B) WP SST, (C) EP SST, and (D) IO SST. The high indexes are calculated using Z850 from 20CR during 1920-2010.


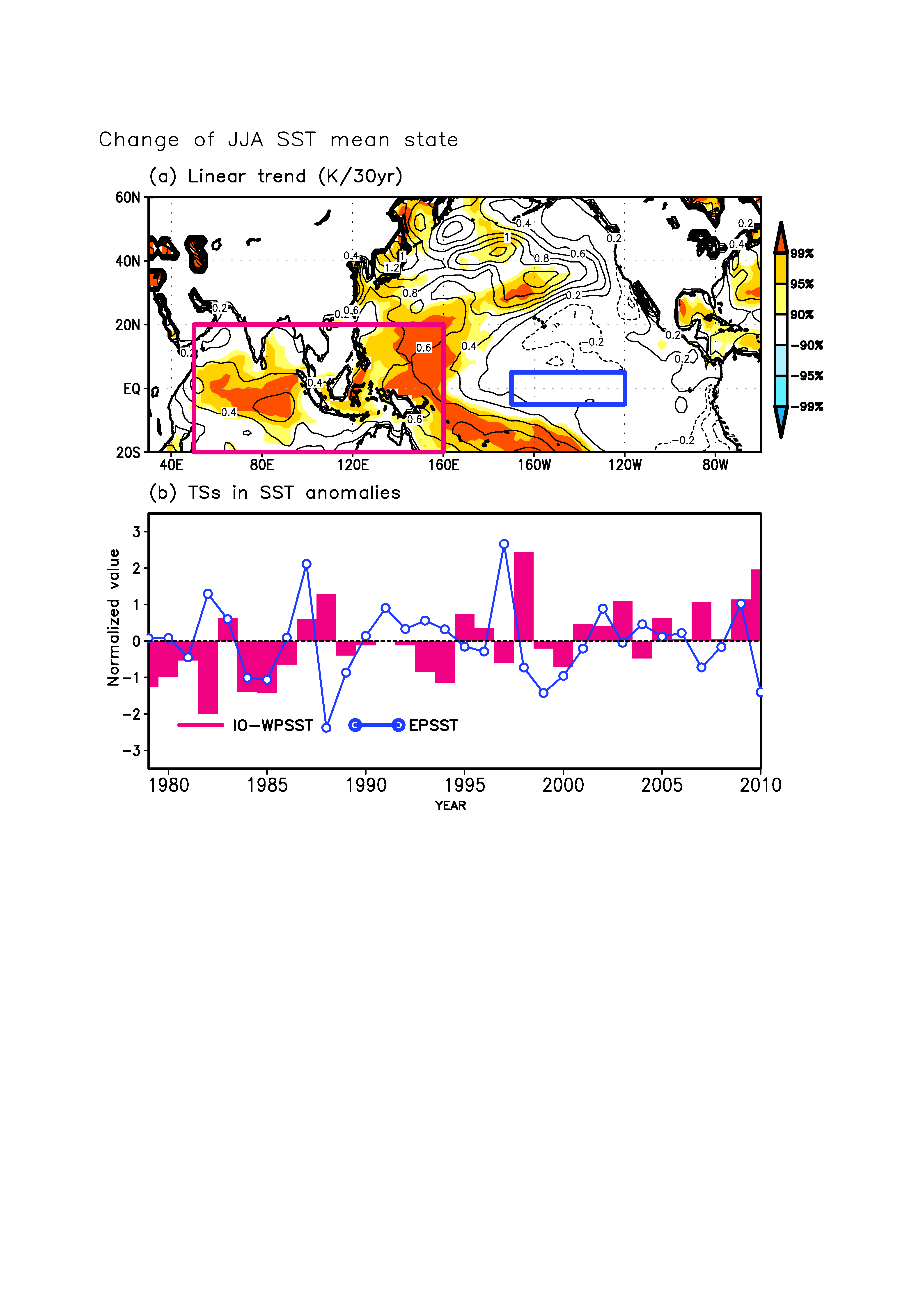


**Figure S5.** (a) Linear trend coefficient of JJA SST during 1979-2010 (unit: K/30yr). (b) Timeseries of normalized SST anomalies averaged over IO-WP [20°S-20°N, 50°E-160°E] and EP [5°S-5°N, 170°W-120°W]. Shading in (a) indicates the value significant above 90% confidence level. The map in this figure was drawn using GrADS.


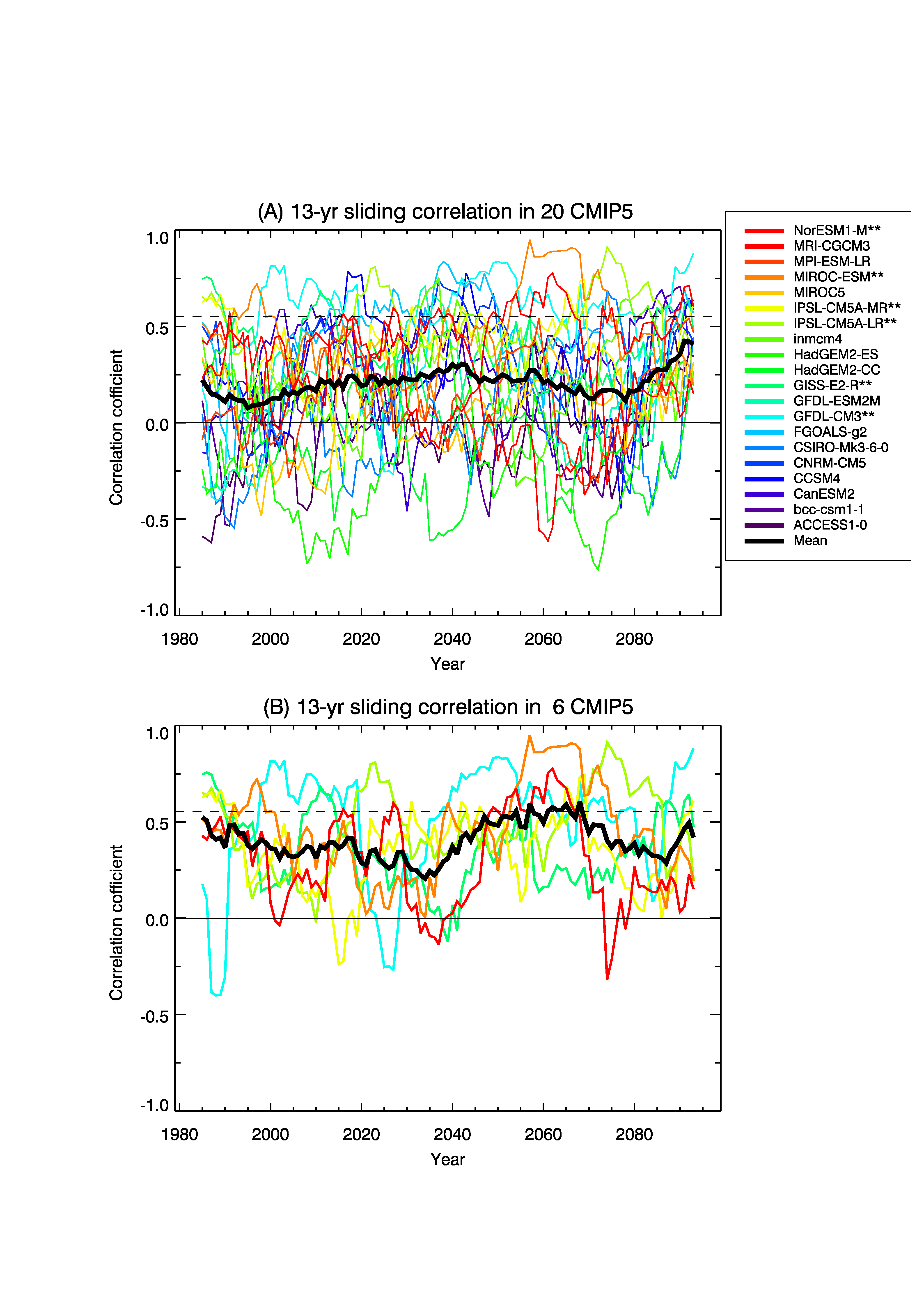


**Figure S6.** (A) 13-yr window sliding correlation coefficient between the NPSH and WNPSH using Z850 for all CMIP5 models during 1979-2099. (B) Same as (A), but for the selected 6 models. The six models are selected for having similar correlation coefficients with that of observation (r ~ 0.5). The thick black line denotes the averaged correlation coefficient for (A) all models and (B) six models.


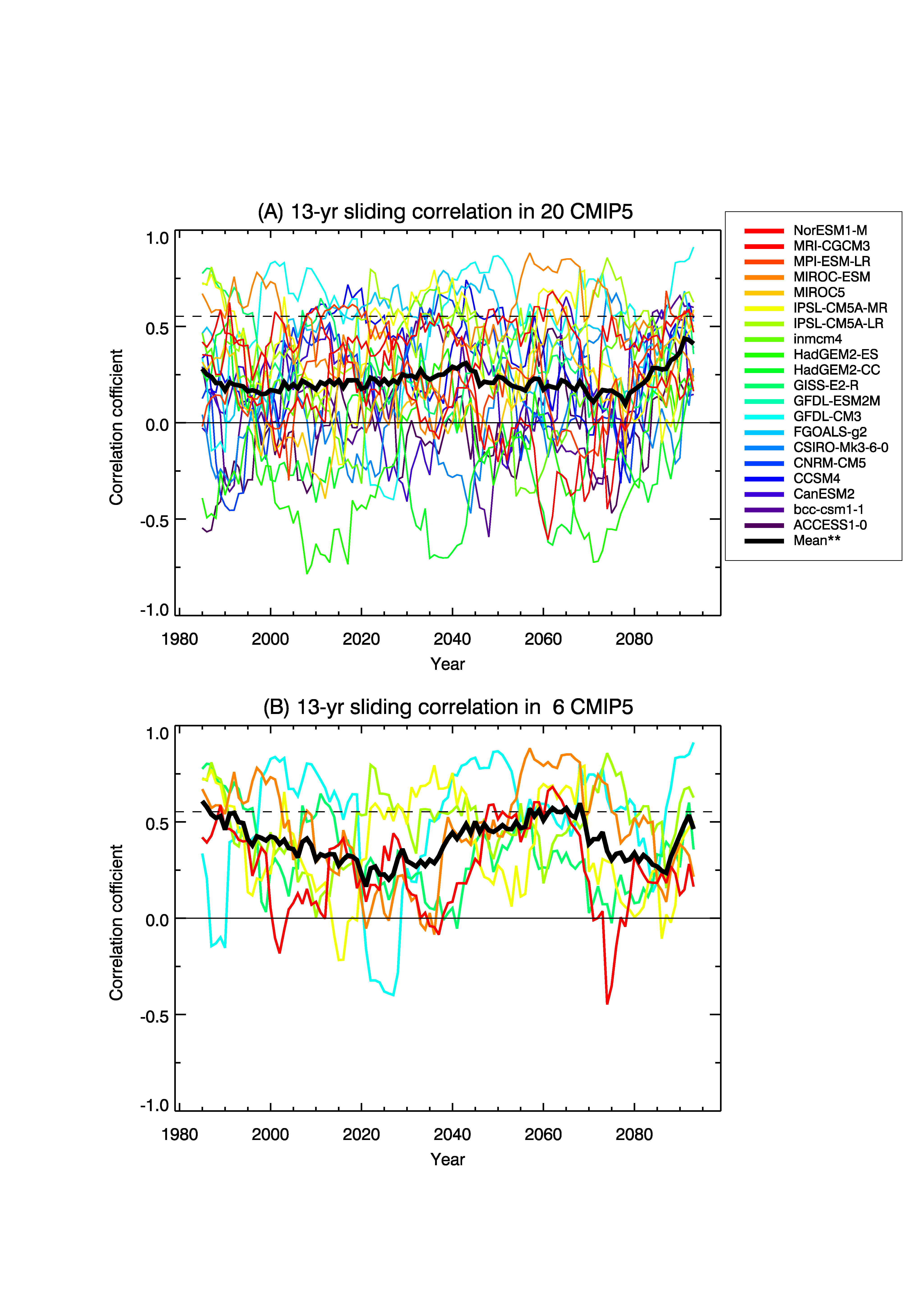


**Figure S7.** Same as Fig. S6, but for the subtropical highs using SLP.


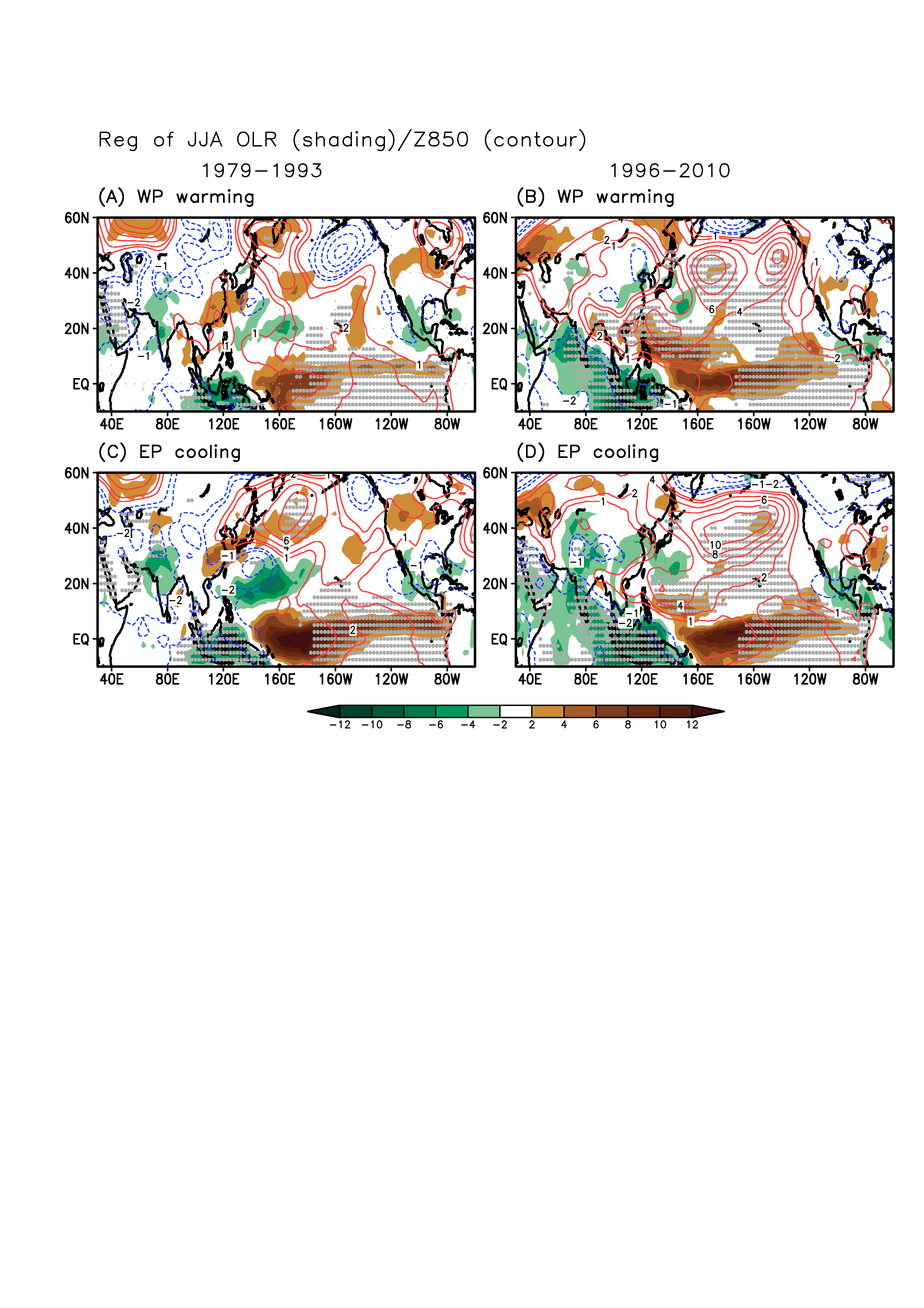


**Figure S8.** Regression of JJA OLR (shading) and Z850 (contour) anomalies. (A) against tropical WP SST time series during 1979-1993; (B) against WP SST time series during 1996-2010; (C) against EP SST time series during 1979-1993; (D) against EP SST time series during 1996-2010. To emphasize the cooling effect of EP SST, the EP SST time series in (C)-(D) are multiplied by -1. The gray dots indicate the Z850 anomalies significant at the 95% confidence level. The map in this figure was drawn using GrADS.


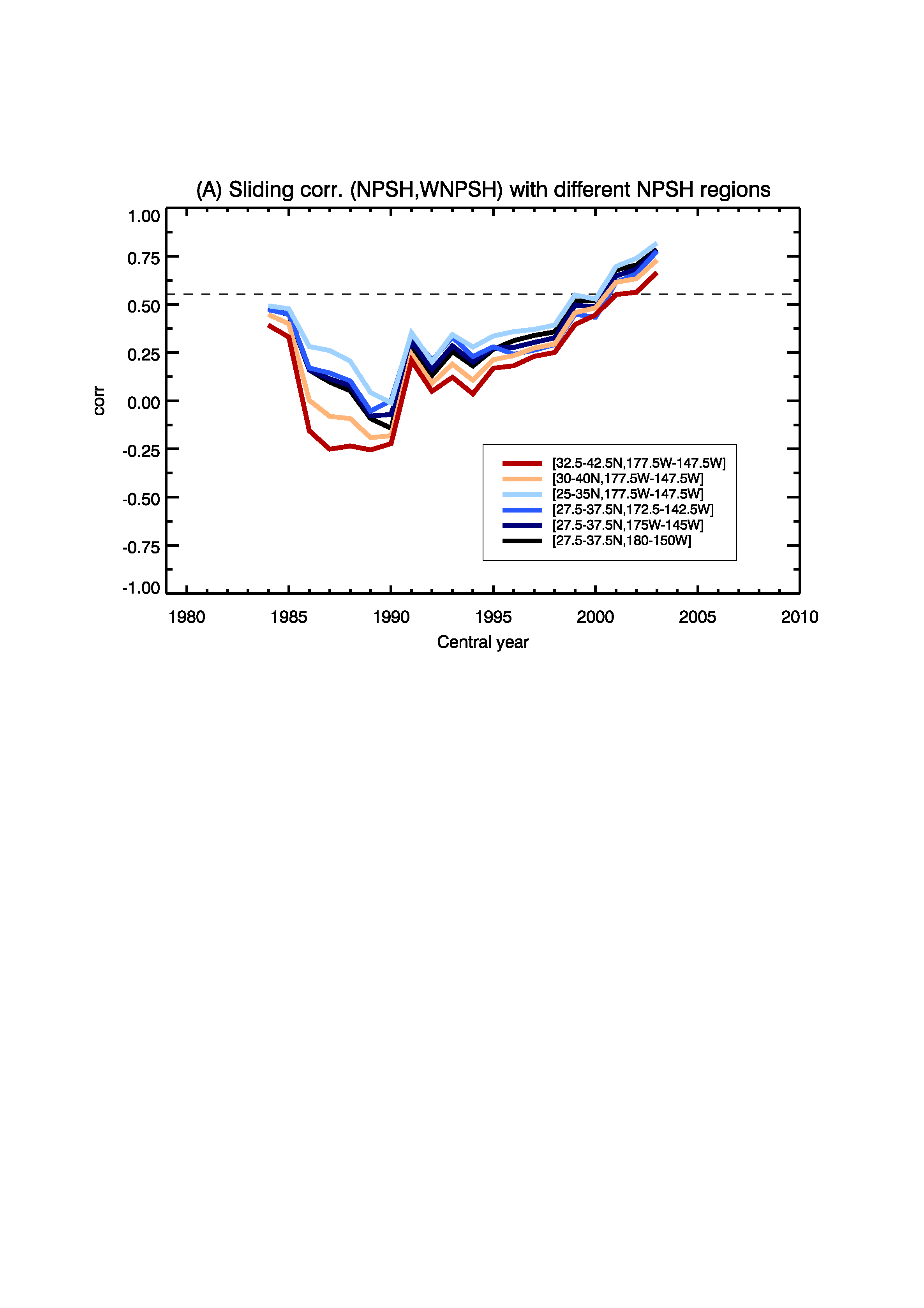


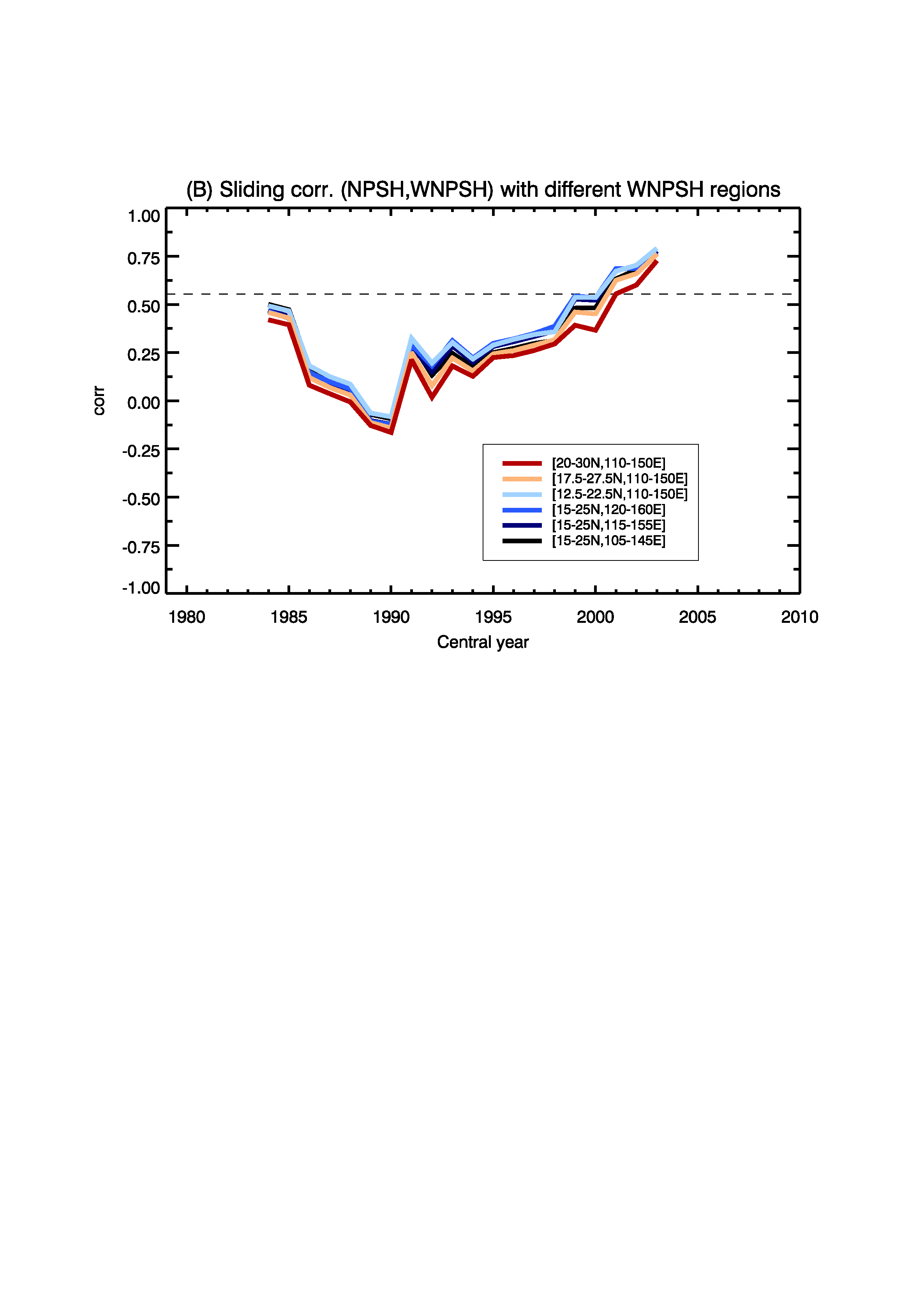


**Figure S9.** 13-yr window sliding correlation coefficient between the NPSH and WNPSH indices with different domains: (A) varying NPSH domain with fixed WNPSH domain; (B) varying WNPSH domain with fixed NPSH domain.


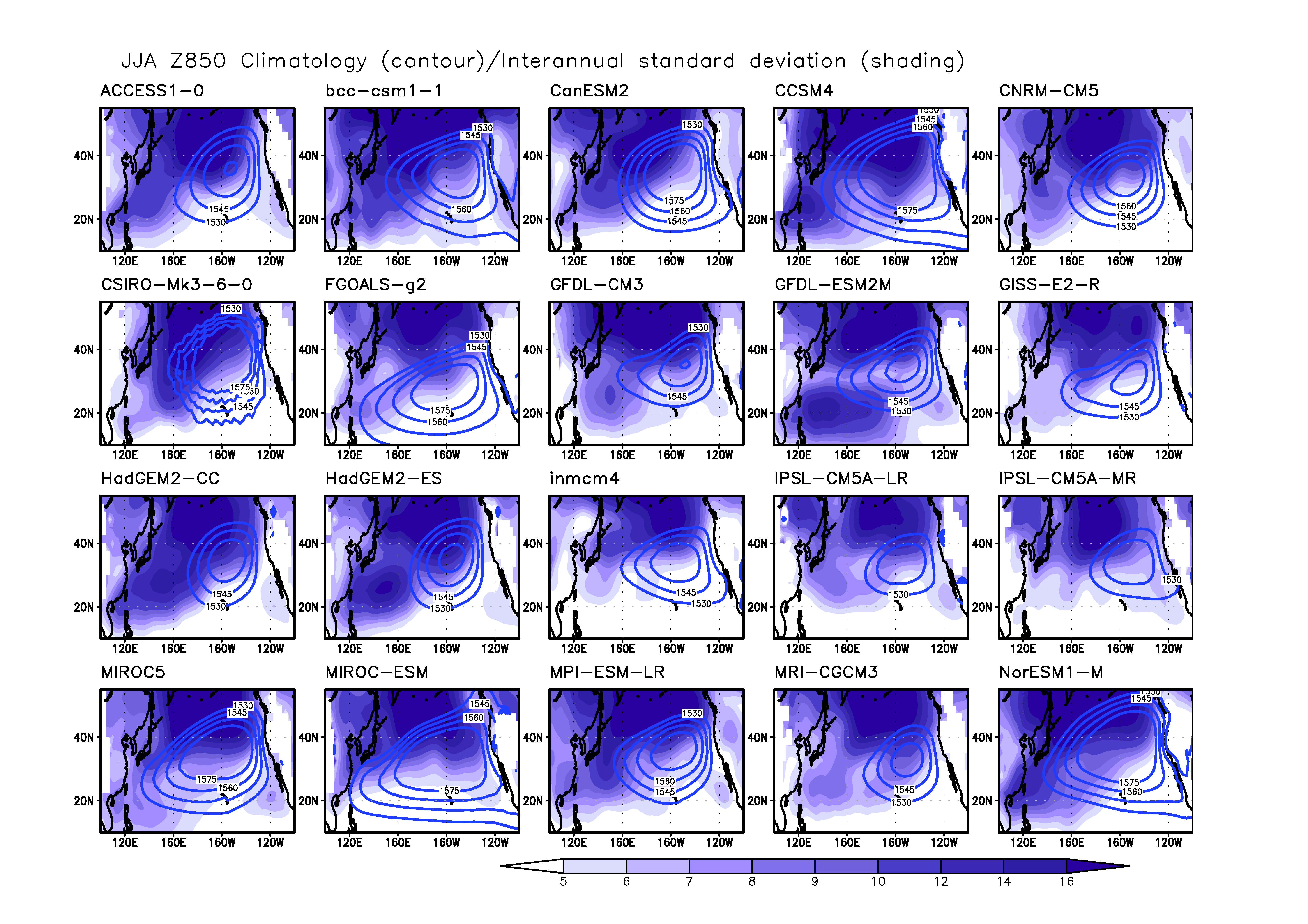


**Figure S10.** Climatology (contour) and interannual standard deviation (shading) of June-July-August geopotential height at 850 hPa during 1979-2005 in individual 20 CMIP5 models. The map in this figure was drawn using GrADS.


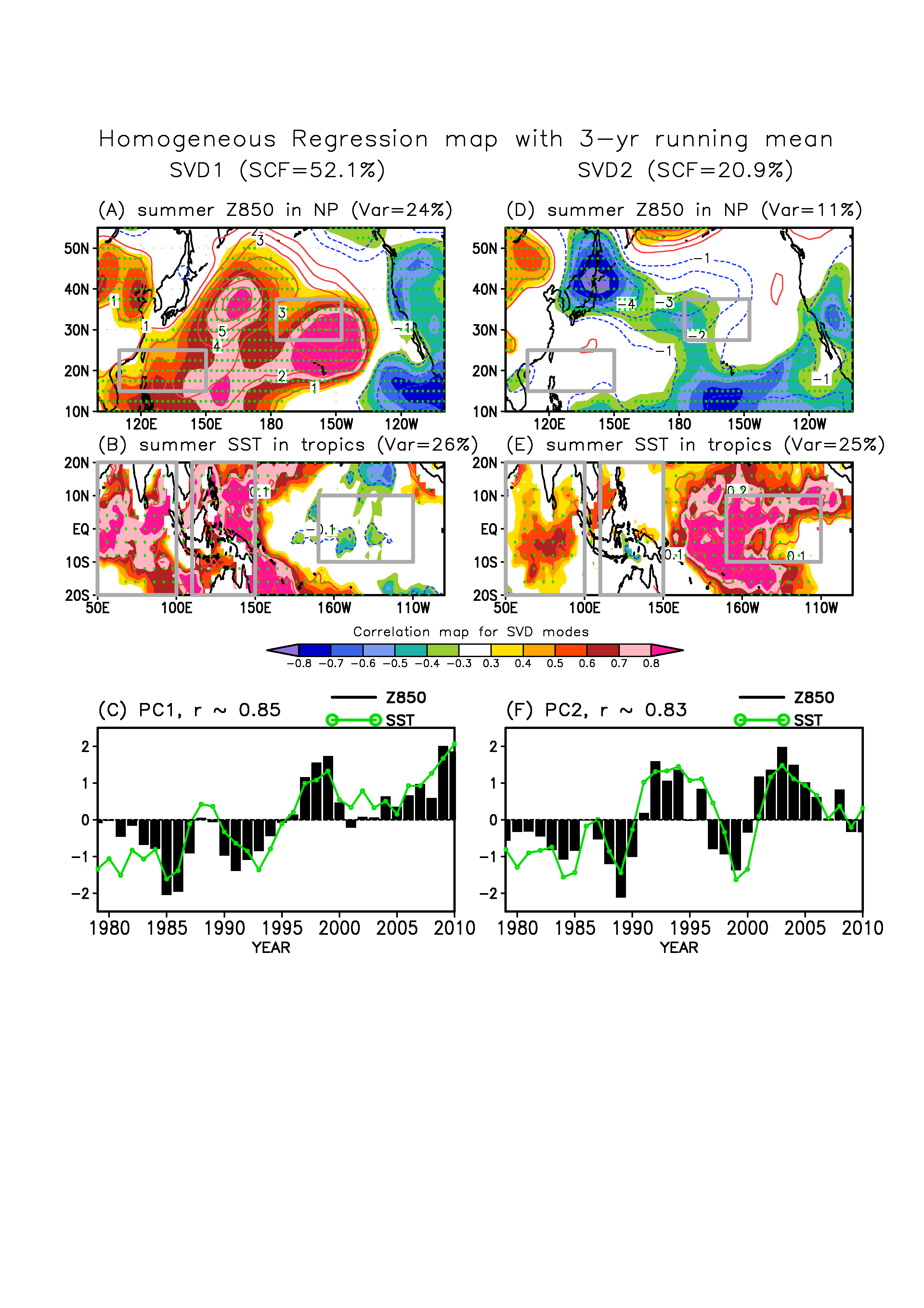


**Figure S11.** Same as Fig. 3, except for SST and geopotential height at 850 hPa with 3-yr running mean. The map in this figure was drawn using GrADS.


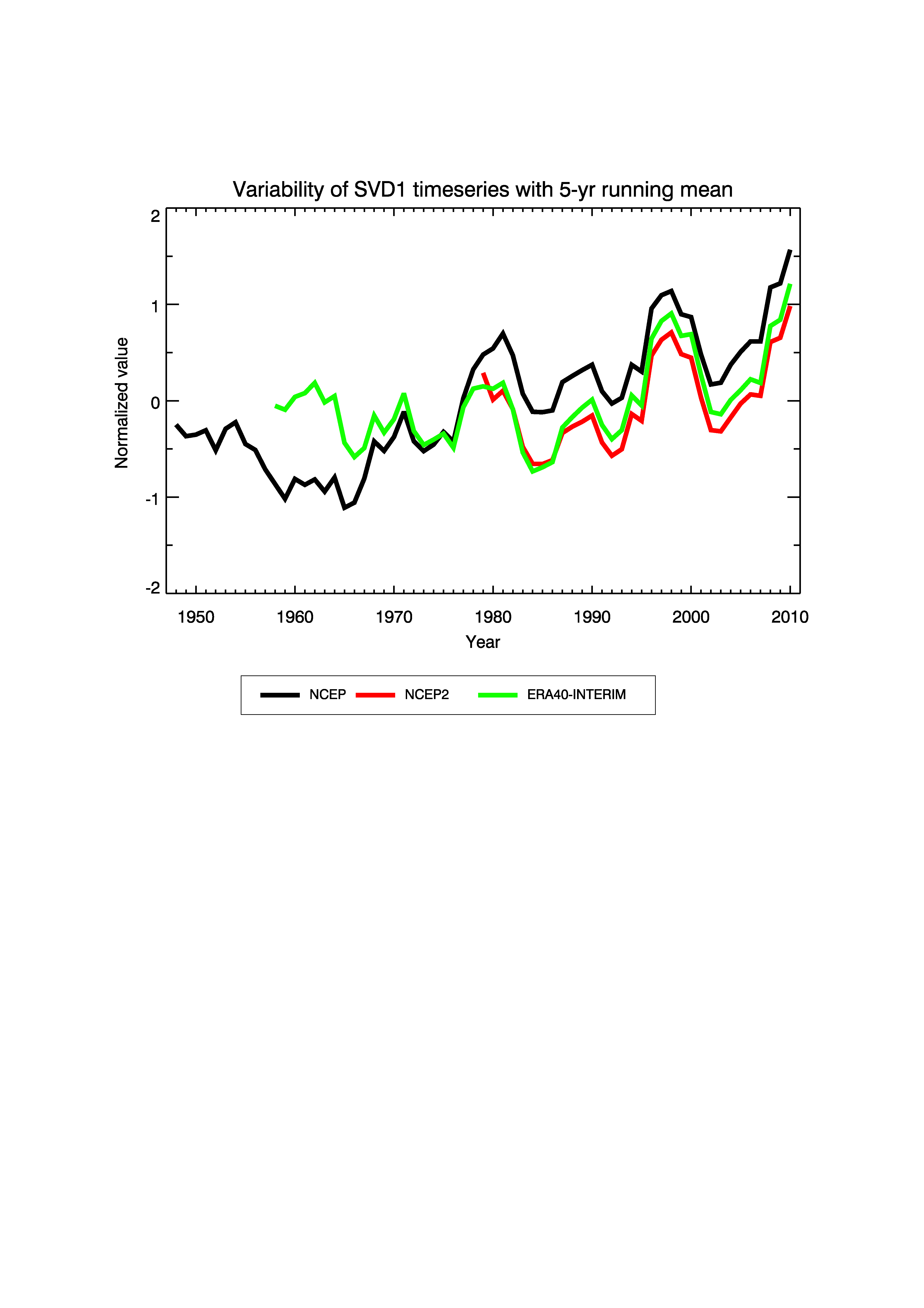


**Figure S12.** Temporal variability of SVD1 PC time series of Z850 with different reanalysis datasets of NCEP (1948-2010), ERA40-Interim (1958-2010), and NCEP2 (1979-2010). A 5-yr moving average was applied to all time series.
